# Comparative Ergot Alkaloid Elaboration by Selected Plectenchymatic Mycelia of *Claviceps purpurea* through Sequential Cycles of Axenic Culture and Plant Parasitism

**DOI:** 10.3390/biology9030041

**Published:** 2020-02-25

**Authors:** Peter Mantle

**Affiliations:** Biochemistry Department, Imperial College, London SW7 2AZ, UK; p.mantle@imperial.ac.uk

**Keywords:** ergot fungi, ergot alkaloids, plectenchymatic morphology, ovary parasitism, axenic culture: ergotamine, lysergic acid

## Abstract

Ergot alkaloids have an established place in plant pathology and toxicology. As pharmaceuticals, their sourcing is via natural or managed agricultural occurrence of sclerotia of *Claviceps purpurea* (Fr.) Tul. or through industrial fermentation processes with other *Claviceps*. The key factor for biosynthesis is differentiation of a particular mycelial anatomy. Previous study of these fungi from two disparate English grass genera, *Spartina* and *Phragmites*, has shown that only mycelia expressing a plectenchymatic sclerotium-like anatomy in specific axenic culture conditions elaborated ergot alkaloids, and then only as far as lysergic acid. The present report describes sequential cycles of axenic and parasitic cultivation for wild isolates from *Dactylis* and *Alopecurus* with intervention of a single ascospore step. This confirms the homozygous character of *C. purpurea* and defines several potential experimental axenic and parasitic conditions within the species for comparing genomic aspects of partial or full biosynthesis of cyclic tri-peptide alkaloids. Whereas *Alopecurus* ergot isolates readily parasitized rye, use of *Dactylis* isolates as inoculum for rye ovaries failed to cause the usual sphacelial fructification but supported growth of exceptionally thin sclerotia, sometimes two in a floret, with low alkaloid content attributed to reduced medullary component. However, after two cycles of axenic and rye-parasitic cultivation, and consistent re-selection of the plectenchymatic character in axenic mycelia, typical growth of ergot sclerotia occurred on rye. Caution thus seems necessary in tests for putative host specificity in any taxonomic realignments within the classical concept of *C. purpurea*. A *Dactylis* ergot isolate was also uniquely shown to parasitise the plumule of germinating rye seeds confirming the susceptibility of apical tissues. A key biosynthetic feature of a mycelial glyceride oil, rich in ricinoleic acid, as a prelude to axenic and parasitic formation of ergot alkaloids by *C. purpurea* is emphasised.

## 1. Introduction

The long history of ergot as a cereal and grass parasite, adversely affecting humans and agricultural ruminants, gradually evolved to include beneficial pharmaceuticals for which specific cultivation on rye on an industrial scale in Central Europe (e.g., in Germany, Czechoslovakia, and Switzerland) was supplemented also by sclerotia salvaged via cereal seed cleaning. In England, in the context of the developments of ergometrine in obstetrics [1], Burroughs, Wellcome & Co processed raw ergot for pharmaceuticals during the first half of the last century and explored agricultural cultivation of *Claviceps purpurea* (Fr.) Tul. The advent of industrial fungal fermentations offered a challenge to mimic the biosynthesis of ergot alkaloids, previously the preserve of parasitic sclerotia. The first significant axenic culture fermentation for ergot alkaloids, by an Italian isolate of *Claviceps paspali,* produced a simple derivative of lysergic acid [2] that became an industrial process. This was followed by Tonolo in the same Rome laboratory by the selection of a *C. purpurea* isolate, collected in Spain from the wheat/rye hybrid Triticale. Following extensive mycelial selection in axenic culture on rich nutrient media [3], a compact plectenchymatic form was selected, perceived as resembling that of the early sclerotial stage in rye florets [4]. That isolate subsequently produced the important cyclic tripeptide alkaloid ergotamine in a 10-day submerged culture fermentation in a 20% mannitol/3% Difco peptone broth pH 6.2 [5] although, as explained subsequently [6], the ~1 mg/mL yield was still too low for commercial exploitation. Ergotamine production was also described concurrently [7] without identity of the fungus; Tonolo (personal communication) assumed it was his strain.

To seek understanding of the general difficulty of mimicking in axenic culture what *C. purpurea* apparently so easily achieves during ovary parasitism of host plants, a search for suitable fungi ensued. Highly selected isolates from English Gramineae, e.g., *Spartina townsendii* H. et J Groves and *Phragmites communis* Trin. [8,9], had an ecology which at least made them of low or no epidemiological risk towards cereal crops. They proved unexpectedly fruitful in expressing in axenic culture a pigmented growth form described as plectenchymatic. The term reflected its compact composition in which cells of component hyphae gradually lose evidence of their filamentous origin, as is evident in longitudinal sections of developing ergot sclerotia. This tissue was characterised also by conforming to the natural anatomy of *C. purpurea* (ergot) sclerotia by their prominent glyceride oils having the hydroxy-fatty acid ricinoleic acid as the principal fatty acid in an estolide conformation [10]. In these estolides, further esterification of a triglyceride intermediatee with other fatty acids leads to triglycerides with 4, 5 or 6 fatty acid substituents. This oil of *C. purpurea* is apparently unique amongst fungi and can be used as a diagnostic indicator for ergot contamination in animal feed [11]. Assumption that *C. purpurea*, as an ascomycete, produces heterocaryotic ascospores was expressed [12,13] in considering variations in parasitic alkaloid-producing ability and stability. Also, there was uncertainty as to whether or not only heterokaryotic mycelia are able to produce alkaloids both in vivo and in vitro. Experimental analysis by Esser and Tudzynski [14] used two commercial pharmaceutical strains in concluding that *C. purpurea* is monoecious, and demonstrated that its whole life cycle could occur under homokaryotic conditions. Nevertheless, a recent review still illustrates the *C. purpurea* life cycle with karyogamy and meiosis within the ascosporogenous stage [15], without citing experimental evidence of reality. A single ascospore phase was therefore incorporated into the present studies with wild ergot isolates to complement those of Esser and Tudzynski, whose commercial strains would have had defined alkaloid pedigrees.

Previously, selected morphology of an isolate of *C. purpurea* from an unusual salt-marsh habitat was found to produce lysergic acid in stationary liquid fermentation [8]. Extension, with ergot from two common agricultural grasses, integrating selection of axenic, plectenchymatic and alkaloid-producing morphology with parasitic performance, was continued through several cycles over several years. The objective was to assess the resilience of sclerotium-like differentiation aided by morphological selection across the two cultural modes, while also including single ascospore selection, to establish axenic potential for the study of alkaloid biosynthesis in *C. purpurea*.

## 2. Materials and Methods

### 2.1. Sources of Experimental Ergot

Ergot sclerotia were collected from *Alopecurus myosuroides* Hudson at the National Institute of Agricultural Botany, Cambridge (Grid reference 433556) and *Dactylis glomerata* L. at a roadside site at Keal Cotes, Lincolnshire (Grid reference 3689 6087). The former is an important weed grass for cereal crops in which it can be a significant factor in ergot epidemiology [16]; sclerotial alkaloid was mainly ergotoxine (0.69%), a mixture of ergocornine, ergocristine and ergocryptine. The latter host grass usually occupies pasture and waste ground; the small sclerotia (1 × 3–6 mm) contained 0.4 % alkaloid, mainly ergotamine. This classical ergotoxine group of alkaloids above differ from each other by one amino acid in the cyclic tripeptide moiety, namely phenylalanine, valine or leucine, respectively. Thin-layer chromatography (TLC) analysis for the present study did not differentiate these constituents, but the ergotoxines’ Rf value was well resolved from that for ergotamine.

### 2.2. Cultivation of Axenic Mycelia

Isolation of the fungus from sclerotial tissues was as previously described [8,9]. Ergots were cut into two or three pieces to expose the medullary tissue, from which new mycelium could arise. The following medium (modified from Stoll et al. [17]) was used throughout these studies, whether in liquid culture (100 mL medium per 500 mL Erlenmeyer flask) or as agar slopes or plates or for initial isolation from natural ergots: sucrose 100 g, asparagine 10 g, Ca(NO_3_)_2_ 1 g, MgSO_4_.7H_2_O 0.25 g, KH_2_PO_4_ 0.25 g, KCL 0.1 g, FeSO_4_.7H_2_O 0.33 g, ZnSO_4_.7H_2_O 0.27 g, yeast extract 0.1 g, cysteine hydrochloride 0.01 g, distilled water 1 L, pH 5.0 adjusted with NH4OH. Liquid cultures and agar plates were grown stationary at 24 °C for two weeks. An additional 2-stage shaken liquid fermentation process used a sucrose-asparagine broth seed stage for 8 days at 24 °C followed by a 10% vol/vol transfer to an alkaloid-production phase (sucrose (10%), citric acid (2%), adjusted to pH 6 with NH4OH) for 14 days. Single ascospore cultures were obtained by allowing sclerotia bearing mature stromata, suspended inverted from a petri-dish lid, to discharge spores on to water agar for a few minutes during lid rotation. The agar was then scanned stereoscopically and single ascospores transferred to nutrient medium on the tip of a tungsten wire sharpened chemically in molten sodium nitrite.

### 2.3. Analysis of Ergot Alkaloids

The descriptor ‘ergot alkaloids’ here refers to the classical group of nitrogen-containing metabolites of *C. purpurea* derived from tryptophan and mevalonic acid. These include ergotamine and the ergotoxine group which are all derived from the same intermediate (lysergic acid) and then linked to a cyclic tripeptide [18]. Also included are clavine alkaloids, derived similarly, but occurring either as a precursor of lysergic acid (e.g., chanoclavine) or as end products. Some clavines are prominent metabolic derivatives in other fungi [19]. The alkaloid content of sclerotia was determined by extracting, five times with di-ethyl ether, a finely ground aliquot which had been mixed to a wet paste with sodium bicarbonate. Combined ether extracts were shaken out with 1 % tartaric acid, and this was assayed colorimetrically by comparing, with a standard alkaloid solution, the blue colour developed after mixing, as above, with van Urk reagent. Alkaloids were resolved by TLC on Silica Gel G with ethyl acetate/ethanol/dimethylformamide (13: 0.1:1.9).

Quantitative colorimetric ergot alkaloid assay was made in liquid culture filtrates after mixing with twice the volume of van Urk reagent (O.125 % p-imethylaminobenzaldehyde in 65 % H2SO4 + 0.1 % of 5 % FeCl3) against a lysergic acid standard. Typical qualitative determination of constituent alkaloids followed the previous protocol applied to axenic culture alkaloids of a plectenchymatic isolate from ergot on *Spartina townsendii* [8]. Briefly, based on earlier structural determination [20], lyophilised culture filtrate was extracted with ammoniacal ethanol. TLC analysis revealed a blue fluorescent component under UV light (350 nm), inseparable from authentic D-lysergic acid as initially recognised in culture filtrate by the method of Abou-Chaar et al. [21]. It co-chromatographed with its isomer Δ8–9 lysergic acid (more recently named as paspalic acid), both of which give a blue reaction after spraying with 3% p-dimethylaminobenzaldehyde in concentrated HCl.

### 2.4. Cultivation of Host Plants

Experimental host plants were Svalofs Fourex spring rye, Svalofs diploid 0201 spring rye, Svalofs tetraploid winter 58424 rye, Triticale winter Russian 1962/607 (Swedish Seed Association, Svalof, Sweden); Petkus diploid spring rye (Plant Breeding Institute, Cambridge, UK); *Secale africanum* Staph (H.G. Schweikardt, University of Pretoria, South Africa); *Secale sylvestre* Host (G. Nakajima, Gumma University, Japan); *Arrhenatherum elatius* L., *Dactylis glomerata* L. (Grassland Research Institute, Hurley, UK). Plants were grown in garden plots or a glasshouse at the Chelsea Physic Garden, London. Inoculation was either by hypodermic syringe injection into cereal florets just before anthesis or by spraying or painting on small grass inflorescences at stigma exsertion [22].

### 2.5. Analysis of Ergot Oils

Glyceride oils extracted from mycelia in vivo and in vitro were Soxlet extracted - with low B.P. petroleum ether and analysed by gas-liquid chromatography [10].

## 3. Results

### 3.1. Ergot from Dactylis glomerata

**Scheme 1:**
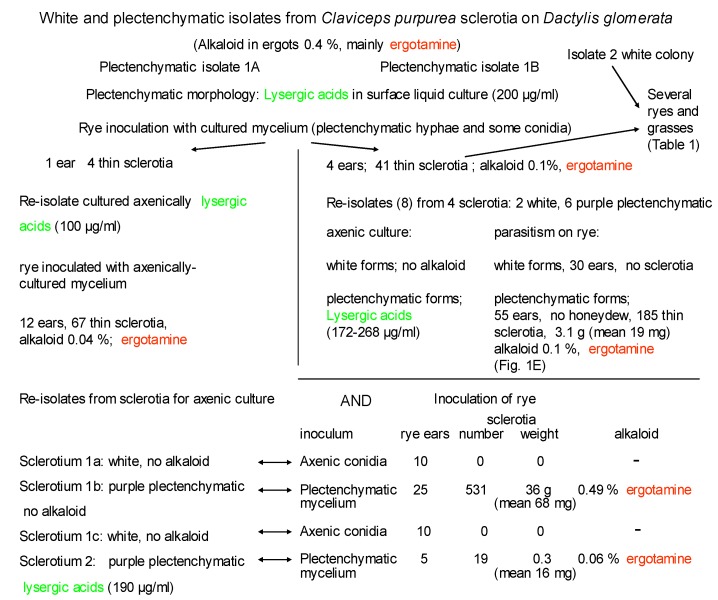
Sequential axenic and parasitic states of ergot from *Dactylis glomerata.*

The experiments, schematically summarised in Scheme 1, follow a sequence of alternating laboratory axenic and host plant parasitic states, with intervening re-isolation from sclerotia to compare growth and alkaloid production performance. Initially, nineteen isolates were made from six sclerotia, selected randomly. During initial isolation, most sclerotia only yielded isolates of white sporulating mycelia on agar cultures (Scheme 1, Table 1) but, from one, isolates from both of two fragments yielded compact plectenchymatic mycelial colonies with pink-purple colouration and some superficial conidial sporulation. Both isolates (A and B) of the latter type were intentionally followed separately, though regarded potentially as replicates. Both isolates formed similar thick plectenchymatic surface mats on stationary liquid culture and a similar yield of alkaloid which consisted mainly of lysergic acids (c.200 μg/mL).

The study of *Dactylis* ergot fresh from the wild commenced with a pair of replicate streams (Scheme 1, mycelia A and B), as far as the simple subdivision of pink-purple plectenchymatic mycelium on agar culture can provide similar/equivalent inocula. Stationary liquid cultures performed similarly, yielding the two lysergic acids (currently described) in the broth beneath surface mats with similar plectenchymatic morphology. Correspondingly, inoculation of rye florets yielded, for stream A, only a few unexpectedly thin sclerotia with ergotamine content much lower (*w*/*w*) than the native sclerotia on *D. glomerata*. Continuing in stream A, re-isolation and selection of mycelia, either white or with a predominantly plectenchymatic morphology, provided inocula for a further cycle of axenic culture and inoculation of rye florets. Notably, white isolates failed both to produce alkaloid in axenic culture and to form parasitic sclerotia. In contrast, one of the plectenchymatic inocula produced abundant large sclerotia on rye, with high content of ergotamine, while another produced much smaller sclerotia containing much less ergotamine.

**Figure 1:**
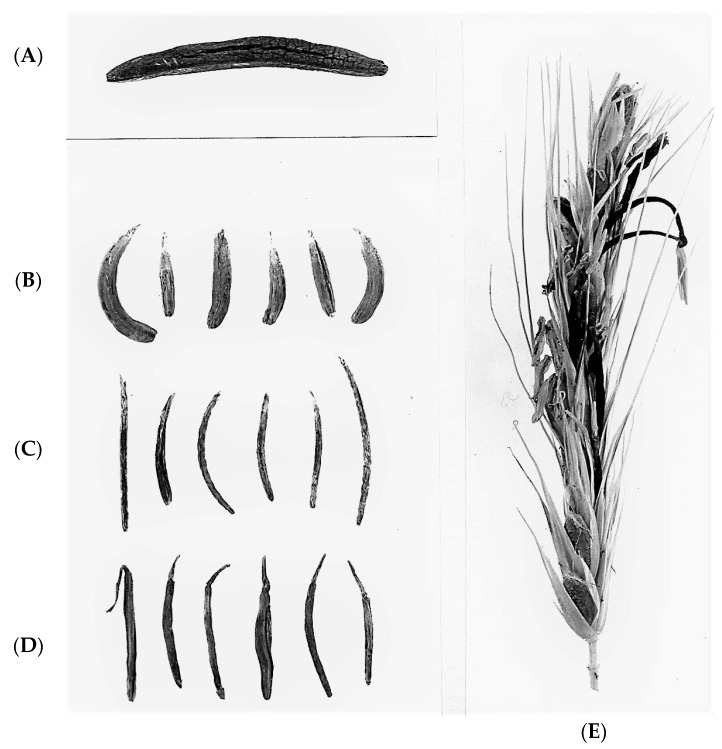
Variations in morphology of ergot sclerotia from different hosts in descending order (**A**–**D**); (**A**), Fourex rye; (**B**), Triticale; (**C**), Petkus rye; (**D**), Winter rye (Scheme 1; Table 1). Sphacelial honeydew exudation was normal early in parasitism, and similar alkaloid composition occurred, in sclerotia of (**A**–**C**). Sclerotia in (**C**) are exceptionally thin, as similarly in (**D**) which had developed in the apparent absence of honeydew and had low alkaloid content. (**D**) sclerotia have anthers retained; plants grown in glasshouse. (**E**); Re-isolate from a thin sclerotium on winter rye (**D**) and inoculated again into winter rye. Developed also in the absence of sphacelial honeydew, and similarly yielded exceptionally thin sclerotia, some of which seem to have split early in development. This resulted in pairs of ergots in florets, free at the base but sometimes fused at the top, and confirmed the exceptionally thin sclerotial morphology. Anthers were retained on sclerotia, since plants were glasshouse grown.

Similarly, in stream B, the pattern of findings replicates the pattern for mycelium A, confirming the capacity of this plectenchymatic inoculum to elaborate lysergic acids in a stationary liquid fermentation. Inoculation of rye florets, on a scale larger than in stream A, demonstrated consistent absence of the honeydew exudate normally associated with the sphacelial stage of the ergot pathogen. However, several sclerotia developed in each rye ear and showed the long thin sclerotia (Figure 1E) as an apparent characteristic of this host/parasite interaction, and with a correspondingly low content of ergotamine.

In contrast, plectenchymatic inoculum derived via a cycle of axenic and parasitic cultures formed ergot sclerotia on all of several ryes and grasses including *D. glomerata* (Table 1) and consistently elaborated ergotoxines as the principal alkaloid. This was instead of ergotamine, and in a typical abundance of 0.23%–0.45%. Sclerotial mass and morphology varied across different hosts, as expected according to the natural vigour of the plants and conformation of their florets, but were notably thin on Petkus rye. Particularly, sclerotia on the winter rye inoculated with the plectenchymatic inoculum (Table 1, PM) were the most abundant and double the mean mass by comparison with those occurring from a conidial inoculum (CC) freshly isolated from wild *Dactylis* ergot.

Contemporary study of a white non-plectenchymatic form (Table 1, inoculum CC), sourced from a different sclerotium in the original ergot collection from *D. glomerata*, was made by inoculating it into inflorescences of a winter rye and *D. glomerata,* either by the usual intra-floret injection of rye before anthesis or by spraying on the grass inflorescences at stigma exsertion. This form parasitized winter rye as thin sclerotia (Figure 1D) with rather low alkaloid content, nevertheless expressing this as ergotamine as in the sclerotia from which *C. purpurea* was first isolated. The example illustrated in Figure 1E, derived from the sclerotium in D, developed also in the absence of sphacelial honeydew, and similarly yielded exceptionally thin sclerotia, some of which seem to have split early in development. This resulted in pairs of ergots in florets, free at the base but sometimes fused at the top, and confirmed the exceptionally thin sclerotial morphology. Anthers were retained on sclerotia since plants were glasshouse-grown. Notably in Table 1, re-parasitism of *D. glomerata* was also ultimately achieved with alkaloid content similar to that in the wild. In conclusion, whereas most of the findings in Scheme 1 concerning mycelia A and B had much in common, outcomes in Table 1 implied that they did not constitute perfect replicates.

**Scheme 2:**
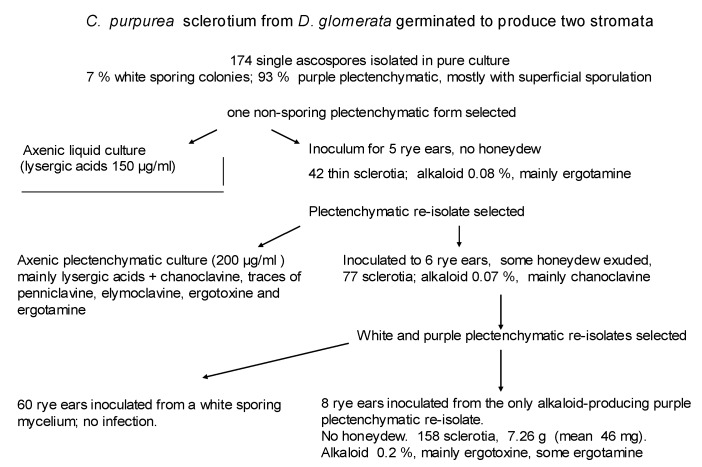
Sequential cultural states from an ascospore isolate from Dactylis ergot.

A study of the influence of single ascospore selection runs in parallel with that in Scheme 1, arising from the same *D*. *glomerata* source. Experimental findings are displayed in Scheme 2. It concerns a rare non-sporing plectenchymatic mycelial form selected from 174 single ascospore colonies. Performance in axenic culture followed the consistent pattern in yielding lysergic acids and, when inoculated into rye florets, again gave thin sclerotia with a low content of ergotamine but without preliminary honeydew exudation. Selecting a monokaryotic form, as predicted for an ascomycete ascospore, had not therefore prevented it from expressing its native capacity for alkaloid biosynthesis in vivo in spite of the differing morphology in florets of an unfamiliar host. Re-isolated plectenchymatic tissue, put into axenic liquid culture, again yielded mainly lysergic acids, but complemented by chanoclavine and traces of other ergot alkaloids penniclavine, elymoclavine, ergotamine, and ergotoxines. The same re-isolate was inoculated into rye, producing a little sphacelial honeydew and later forming long thin sclerotia with a low alkaloid content consisting mainly of chanoclavine (an early biosynthetic intermediate in lysergic acid biosynthesis). However, re-isolation of the plectenchymatic form from one of these thin sclerotia expressed somewhat more complex patterns of alkaloid products, including chanoclavine, in both axenic and parasitic culture. Further reisolates from sclerotia were almost all white sporulating forms which failed to produce sclerotia in rye. In contrast, a rare purple plectenchymatic one apparently failed to exude honeydew from rye, but nevertheless elaborated abundant sclerotia, not so thin as previously and with normal alkaloid content (0.2%). However, alkaloid end-point profile, mainly ergotoxines and ergotamine, might indicate evolution in biosynthetic selectivity for particular cyclic tripeptide derivatives of lysergic acid. Nevertheless, rather typical *C. purpurea* was achieved, but only after three cycles of host-passaging and cultural morphology selection from this wild ergot of *D. glomerata*.

However, adding the findings from 240 single-ascospore isolates derived from another sclerotium from the same source, amongst which no similarly plectenchymatic form occurred, only emphasises its rarity in the present study. A notable feature in this scheme was occurrence of chanoclavine in axenic culture amongst an array of clavines and lysergyl tripeptides, and also as the principal component of low alkaloid content in these parasitic sclerotia. This is consistent not only with chanoclavine’s early place in ergot alkaloid biosynthesis, but also a paucity of alkaloid in unusually thin sclerotia.

### 3.2. The Seedling Plumule Apex As a Putative Site for Ergot Parasitism

**Figure 2:**
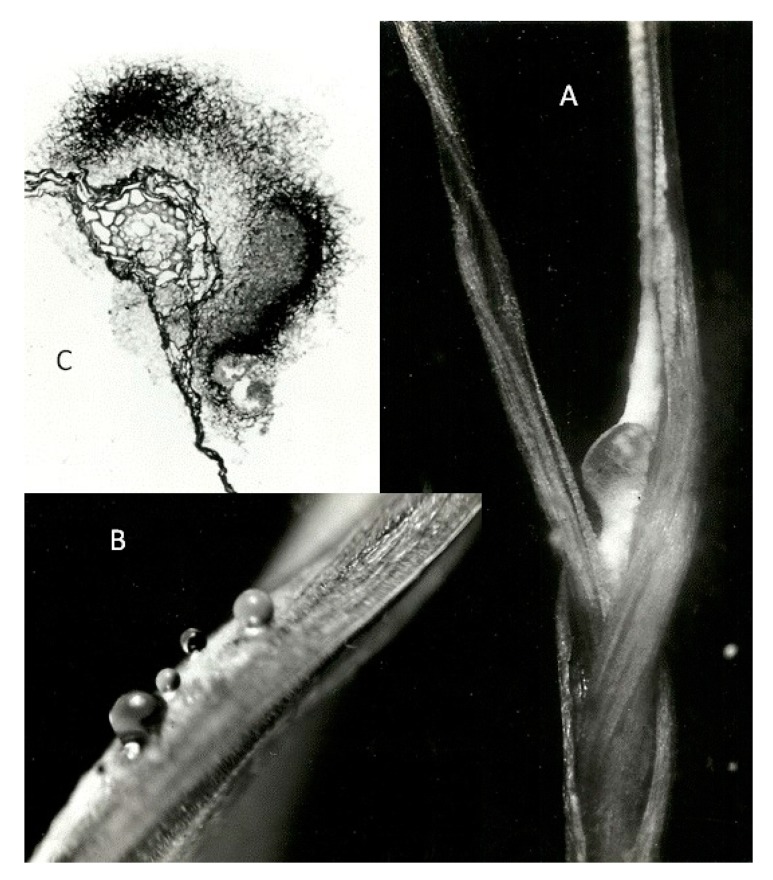
*C. purpurea* infection in an expanding leaf 3 weeks after inoculating the plumule of a germinating rye seed: (**A**) lateral view of dark pigmented lesion on the first leaf emerging from the coleoptile; (**B**) honeydew exudate associated with the white hyphae of sphacelial fructification on the first leaf; (**C**) unstained transverse section of leaf through ‘ergot’ lesion showing dark pigmented fungal cortex and a sphacelial sorus surrounding the leaf’s vascular tract.

Although the unfertilised ovary is the only accessible natural parasitic site of cereals and grasses for ergot fungi, a question arises as to whether undifferentiated apical tissue of a germinating seed can also support parasitism. Sixty surface-sterilised rye seeds, germinated at 18 °C on water agar for three days, were given intra-coleoptile injection of conidial/ mycelial fragments from axenically cultured plectenchymatic mycelium of *Dactylis* ergot grown on sucrose/ asparagine agar. After 10 days at 24 °C, half of the seedlings showed signs of infection in young leaf tissue. After 20 days several lesions had dark-pigmented sclerotium-like areas (Figure 2). It was concluded that the differentiating plumule could support parasitism by *C. purpurea*, expressed as both sphacelial and simple sclerotial components, if inoculum could be placed artificially near the apical growing point without significant collateral tissue damage.

### 3.3. Ergot from Alopecurus myosuroides

**Figure 3:**
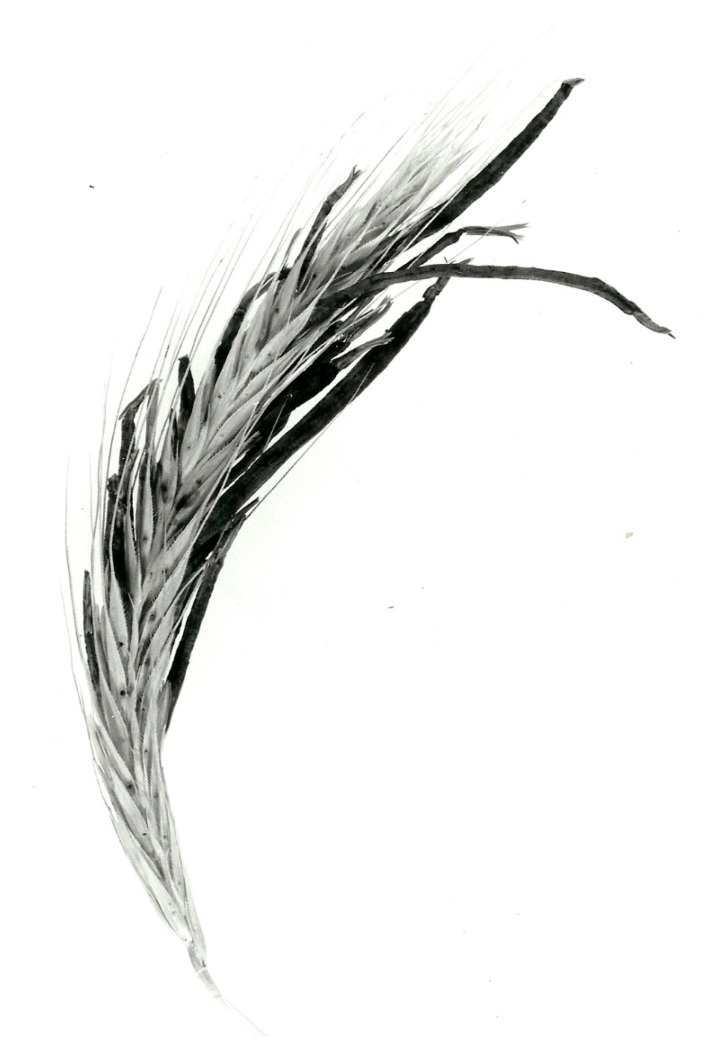
Fourex rye ear bearing sclerotia of *C. purpurea* isolated from ergots on *A. myosuroides*. Glasshouse grown plant inoculated before anthesis; hypodermic needle lesions are on glumes. Circumstances of infected anther filament (and anther) inclusion within developing sphacelium ensured retention of anthers at sclerotial tips.

In contrast to the study with *Dactylis* ergot, the host/parasite outcomes of which could not have been predicted, ergot sclerotia from *Alopecurus* were expected to illustrate a norm for *C. purpurea* as a potential cereal pathogen. The experiment was therefore intended partly as a control for whatever was found for *Dactylis* ergot. The sequence of parasitic and axenic steps with a *C. purpurea* isolate from a sclerotium on *A*. *myosuroides* commenced with demonstration of typical parasitism on Fourex rye (Figure 3) during which ergotoxines were again the principal alkaloid.

**Scheme 3:**
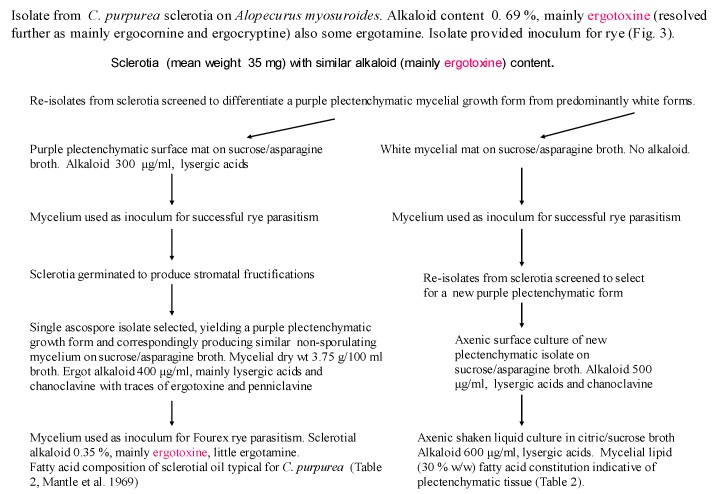
Sequential states for ergot from *Alopecurus myosuroides.*

To find amongst the predominantly white forms a purple plectenchymatic form in axenic culture could not have been expected. However, after extensive selection and sub-culturing, one such form was found (Scheme 3, left track) and it produced lysergic acids. This isolate then produced typical sclerotia on rye. From those sclerotia the ascosporogenous stage was readily produced after appropriate cold-conditioning and a plectenchymatic mono-ascospore reisolate was selected; it grew in the plectenchymatic way on sucrose/asparagine broth, with biomass cytology predictive of being lipid-rich and producing an array of ergot alkaloids which was somewhat more complex than the usual lysergic acids in the *Dactylis* study. Mycelial inoculum from this axenic culture readily produced sclerotia on rye, with an alkaloid content similar to that of the original ergot collection from *A*. *myosuroides,* and a sclerotial oil composition typical of *C. purpurea* (Table 2, [10]). This again demonstrated the monokaryon’s character in retaining both pathogenic and alkaloid-synthesis capacities.

Considering the experimental sequence with white mycelial forms (Scheme 3, right track), an example successfully parasitized rye and a plectenchymatic form was selected from among re-isolates from the sclerotia. In the usual axenic culture on sucrose-asparagine broth, it produced alkaloid, mainly lysergic acids and chanoclavine. This form also produced lysergic acids in submerged fermentation (Table 2). This fermentation supported a suspension of plectenchymatic hyphal fragments swollen on account of lipid revealed histologically by Sudan 3 staining. The fatty acid composition of the mycelial oil closely resembled that for the oil in *C. purpurea* sclerotial tissue (Table 2). The composition also corresponded closely with that from a repeat of a former ergotamine-producing fermentation (Tonolo 1966), particularly concerning the hydroxy-fatty acid, ricinoleic acid (Table 2, OH-18-1).

## 4. Discussion

Generally, for both sources of ergot, elaboration of ergot alkaloids in axenic culture was possible, even if challenging to select the appropriate mycelial morphology via multiple subculturing and observation. For the *Dactylis* source, white sporing morphology was nonpathogenic but the plectenchymatic form became parasitic in rye inflorescences, even if appearing that this was an abnormal host. The *Alopecurus* source served well as a control for the generally weak pathogenicity of the *Dactylis* ergot isolates towards rye. More specifically, the studies have demonstrated that wild *C. purpurea* from *A. myosuroides* could readily parasitizes rye. Also, a mycelial form expressing a plectenchymatic structure could operate the ergot alkaloid biosynthetic pathway as far as the lysergic acid intermediate of the cyclic tripeptide alkaloids typically found otherwise in the fungus’ plant-parasitic sclerotia. In comparison, the ergot pathogen from *D. glomerata* could similarly be amenable to selection of a plectenchymatic growth form, also performing biosynthesis of lysergic acid, but notably weak in rye parasitism, supporting unusually thin and sometimes split sclerotia with low alkaloid content. Axenic culture findings mimic those for ergot from *Spartina* and *Phragmites* grasses. The *Spartina* ergot isolates [8] had exhibited very weak parasitism of rye; only a few cryptic aborted bodies were found. However, after reisolation from them, selection of a plectenchymatic form in axenic culture, which produced lysergic acids, caused abundant sclerotia on rye, typical of *C. purpurea*. Those sclerotia had also typically produced ascospores. Somewhat similarly, the *Phragmites* isolates had also only weakly parasitised rye, abnormality being a lateral attachment to an aborted seed where the sclerotial tissue replaced the embryo side of the seed. This suggested that the parasite had only partly invaded the unfertilised ovary and had developed sclerotial tissue while the uninfected ovary sector differentiated into an incomplete seed [9]. More extreme aberration was recorded where a viable seed developed distal to a sclerotium in a rye floret [23], further illustrating unconventional occurrences in *C. purpurea* parasitism. However, for the present ergot from *D. glomerata* after selection of a plectenchymatic form in axenic culture and successive parasitic passaging, the fungus performed typically as for *C. purpurea*.

Contrasting findings across a range of putative hosts in Table 1 are not easy to explain, but might have reflected the generally greater accumulation of alkaloid in the more conventionally shaped ergots in the ryes, as shown by their mean sclerotial weight. A striking feature of some of these parasite/host interactions is the unusual shape of sclerotia. In some hosts the dimensions are much distorted, being both much thinner and longer than expected (Figure 1). Hence, Petkus and Fourex ryes are both tetraploid, but ergot sclerotial conformations contrast markedly. Triticale, as a wheat/rye hybrid, also has potential for supporting a bulky seed and expectations of a similarly bulky ergot form; expectations were fulfilled (Figure 1B). The *Secale*, *Arrenatherum,* and *Dactylis* species all have small florets and ergots were correspondingly small. The unexpected dominance of ergotoxines in sclerotia from the plectenchymatic inoculum (Table 1), in contrast with that of ergotamine in sclerotia from the white conidial inoculum derived from a separate *Dactylis* ergot sclerotium, might indicate heterogeneity of the wild *C. purpurea* population on this grass. However, in a direct comparison on the winter rye 58424, a marked difference was noted in mass and alkaloid between ergots from plectenchymatic and conidial inocula of the same isolate. This points clearly to a difference in inoculum, which remains undefined. In retrospect, study of the cortical versus medullary tissue ratios in transverse section of thin and thick sclerotia could have shown that thin sclerotia were disadvantaged by proportionately less alkaloid-synthesising cortical biomass.

Molecular genetics studies have revealed distinct species hidden within the classical concept of *C. purpurea* [24], distinguished also by Continental European native host range and ecology. Thus *C. spartinae* is assigned for *Spartina townsendii* ergot. Ergot on *Phragmites communis* was authenticated as the old descriptor *C. microcephala* (Wallr.) Tul., but a new name (*C. arundinis*) has been proposed [24]. Notably, the *C. microcephala* descriptor had previously been considered [9]. Finally, *C. purpurea sensu stricto* might be applied to the present study for the isolate from *A. myosuroides*, since this grass is a cereal crop weed. Thus, three of the four recently proposed native components of classical *C. purpurea* have now been studied across a 200 mile transect in England. In this context, it is notable that the new ergot alkaloid, purpurolic acid [25], was first recognised in *C. purpurea* from *A. myosuroides* [26] which in the present study is seen as *C. purpurea sensu stricto*. That the structure of purpurolic acid was determined, after isolation from an organic acids fraction from ergot in Canadian wheat cleanings, adds some support to the present finding that English *A. myosuroides* ergot conforms to a classical concept of *C. purpurea.* It seems that wheat and rye are similarly susceptible to each other’s natural *C. purpurea* pathogen.

Thus, whereas current trends in molecular taxonomy are influencing concepts of speciation, differentiation on the basis of host range between some forms of ergot in UK may need rigourous assessment before taxonomic revisions are contemplated further. The present study on *Dactylis* ergot, with its very thin long sclerotia when first given opportunity to parasitise rye ovaries, places it intermediate within accumulated host-parasite studies for UK, considering that some UK grass ergot populations have readily parasitised rye, producing bulky sclerotia, while others have failed.

The present finding of rye seedling stem apex susceptibility to *C. purpurea* inoculum, when introduced locally during early germination, demonstrates that the pathogen can parasitise any unprotected meristem-like tissue. Normally, during grass and cereal growth, the stem apex is inaccessible, leaving an unfertilised ovary as the only feasible port of entry, either via the stigma by mimicking pollen tubes or by direct penetration through the ovary wall [27,28]. Biochemical mechanisms for gentle avoidance of the host‘s wound reaction at the narrow ovary/rachilla junction, involving secretion of β-glucanase and pectinase, have been demonstrated experimentally [29]. Recent speculation [30] concerns somewhat anthropomorphic host–parasite interactions between genomic factors for a perceived ‘strict organ specificity’ of the rye ovary for *C. purpurea* attack. This assumes authors were unaware that the parasitic mechanism had been addressed experimentally about 40 years ago, in the light of the current knowledge of enzymology of the pathogen’s utilisation of exuded sucrose in honeydew [31]. 

It was also suggested [30] that ergot alkaloids could confer protective benefit towards the host. However, a UK case of ergotism in grazing cattle [32] implies that not only the *Lolium perenne* L. (perennial ryegrass) host to *C. purpurea* became diseased but, since the ergot pathogen’s sclerotia were consumed by cattle, viability of the ergots probably suffered as well. In studies on ryegrass ergots collected from the same pasture a few months later [22], one isolate displayed strong pathogenicity in rye through two infection cycles, maintaining ergotamine as the dominant alkaloid that had previously caused ergotism in the cattle. From another sclerotium, a similar performance occurred in the first experimental cycle. However, while retaining pathogenicity through three more cycles, alkaloid content became decimated to become evident only as chanoclavine, normally regarded as an early biosynthetic intermediate for ergotamine. Nevertheless, sclerotia of the last cycle were still able to support ascosporogenous fructification. Notably, the present occurrence also of chanoclavine as the only accumulating alkaloid endpoint at stages in cycles of rye parasitism for *Dactylis* ergot (Scheme 1) could open opportunity for biosynthetic study in ergots from yet another native grass. 

However, chanoclavine accumulation here may be more a consequence of the pathogen’s difficult attempts to parasitise unfamiliar host ovaries following artificial experimental inoculation of florets when they are particularly susceptible. Notably, the resulting sclerotia were unexpectedly thin. 

In recent years, natural focus on molecular aspects of ergot alkaloid biosynthesis has received much attention, not least because of the expanding number of genetic analysis techniques [18]. However, there remains mystery about the apparent reluctance of *C. purpurea* to operate the alkaloid pathway in vitro, in spite of vast potential for formulating an artificial nutritious environment to allow operation of the biosynthetic gene expression in a parasitizing sclerotium. 

*C. purpurea* without its alkaloids becomes merely an agricultural nuisance. However, attention might be given to its unique estolide oil, rich in ricinoleate, as an experimental indicator of sclerotial differentiation in this species. Even the oil in stromata has no ricinoleate and sclerotia of African *C. sulcata* Langdon also had none [33]. Seedling plumule potential for the study of parasitism might facilitate a laboratory-scale comparison of gene expression for lipid biosynthesis in axenic and parasitic modes using ricinoleate as a marker for sclerotial differentiation. The maintenance of axenic culture expression of selected plectenchymatic morphology of UK wild-grass ergot has proven a challenge without dedicated cultural focus. However, herbarium material of *Phragmites communis* inflorescence bearing small sclerotia of *C. purpurea* has recently been sourced from exactly the same location as collection 1 in 1964 (Grid reference TF 2423, [9]), describing novel plectenchymatic ergot isolates producing alkaloids in axenic culture. This analogous material is available on request.

## Figures and Tables

**Table 1:** Parasitic performance of *C. purpurea* from *D. glomerata* in potential rye and grass host inflorescences. Influence of inoculum (Scheme 1, sourced via plectenchyma selection) from honeydew resident on sclerotia from rye (PM). For contrast outcome in sclerotial yield and alkaloid content, conidia-rich white non-plectenchymatic axenically-cultured mycelium (CC) was obtained directly from the original wild ergots.

Host Plant	Inoculum	Infloresc-Ences Inoculated	Total Ergots	Ergot		Principal Alkaloid (% *w*/*w*)
				Weight (g)	Mean (mg)	
Fourex rye	PM	48	310 Figure 2a	26.35	85	0.34 ergotoxines
Winter rye	PM	48	988	35.93	36	0.24 ergotoxines
Petkus rye	PM	1	43 * Figure 2c	1.2	28	0.23 ergotoxines
*S. sylvestre*	PM	2	22	0.25	11	0.25 ergotoxines
*S. africanum*	PM	4	17	0.42	25	0.28 ergotoxines
Triticale	PM	1	19 Figure 2b	1.2	63	0.30 ergotoxines
*A. elatius*	PM	13	50	0.64	13	0.45 ergotoxines
*D. glomerata*	PM	6	114	0.54	5	0.29 ergotoxines
Winter rye	CC	18	138 * Figure 2d	1.90	14	0.08 ergotamine
*D. glomerata*	CC	Many	2500	10.25	4	0.08 ergotamine

* exceptionally thin sclerotia.

**Table 2:** Fatty acid composition (%) of *C. purpurea* glyceride oils of the isolate from *A. myosuroides*, having similar composition in parasitic sclerotia and plectenchymatic mycelium in axenic fermentation. Included for reference are data from concurrent analysis of fermentation mycelium of the Tonolo 1966 strain in alkaloid-producing and non-producing modes, noting absence of ricinoleate in the latter.

	14:0	16:0	16:1	18:0	18:1	18:2	18:3	OH-18:1
***A. myosuroides* isolate**								
**Parasitic sclerotia on rye**	0.3	27.8	4.2	2.7	20.5	7.0	0.7	36.8
**Axenic plectenchyma in submerged fermentation**	0.2	23.0	2.7	6.5	20.6	13.7	3.2	30.1
**Tonolo 1966 isolate**								
**Submerged. Alkaloid +ve**	0.1	13.2	5.2	1.3	25.1	30.9	0.7	23.5
